# Co-circulation of two envelope variants for both gorilla and chimpanzee Simian Foamy Virus strains among humans and apes living in Central Africa

**DOI:** 10.1186/1742-4690-12-S1-P82

**Published:** 2015-08-28

**Authors:** Léa Richard, Rejane Rua, Edouard Betsem, Augustin Mouinga-Ondémé, Mirdad Kazanji, Eric Leroy, Florence Buseyne, Philippe V Afonso, Antoine Gessain

**Affiliations:** 1 Unit of Epidemiology and Physiopathology of Oncogenic Viruses, Institut Pasteur, Paris, France; 2 Centre National de la Recherche Scientifique (CNRS), UMR 3569, Paris, France; 3 Université Paris Diderot, Cellule Pasteur, Paris, France; 4 Faculty of Medicine and Biomedical Sciences, University of Yaounde I, Yaounde, Cameroon; 5 Unité de rétrovirologie, Centre International de Recherches Médicales de Franceville, Franceville, Gabon; 6 Unité des Maladies Virales Emergentes, Centre International de Recherches Médicales de Franceville, Franceville, Gabon

## 

Simian foamy virus (SFV) is a retrovirus ubiquitous in non-human primates (NHPs) that can be transmitted to humans, mostly through severe bites. In the past few years, our laboratory identified more than 50 hunters from Central Africa infected with zoonotic SFVs. Analysis of SFV complete sequences obtained from 5 of these individuals had revealed that the env gene was the most variable one. Furthermore, recombinant SFV strains have been recently shown; some of them involved the env gene. This led us to investigate the env gene variability of zoonotic SFV strains, looking especially for possible recombinants. We sequenced the complete or the surface glycoprotein region (SU) of SFV env gene amplified from blood DNA of: 1) a series of 40 individuals from Cameroon or Gabon infected with a gorilla or chimpanzee FV strain; 2) one gorilla and 3 infected chimpanzees living in the same areas than the hunters. All sequences were aligned and analysed by phylogenetic (neighbour-joining and maximum likelihood) and recombinant detection methods (similarity plot and bootscan analysis, Recombination Detection Program). Phylogenetic analyses revealed the existence of two different env variants among both gorilla FV and chimpanzee FV strains. These were present in zoonotic as well as in NHP strains. These variants differ greatly (more than 30% variability) in a 750 bp long region located in the receptor binding domain of the SU; the rest of the gene is very conserved (less than 5% variability among strains from the same species). Within a given variant, protein sequences of the SU are very conserved and stable (non-synonymous/synonymous mutations ratio <0,1) which may reflect functional constraint. Recombination analyses highlighted that these variants could have emerged through recombination events.

